# Einsatz von KI im Versicherungssektor – mit Schwerpunkt Versicherungsmedizin

**DOI:** 10.1007/s12297-022-00527-2

**Published:** 2022-07-26

**Authors:** Christian Armbrüster, Jonathan Prill

**Affiliations:** Fachbereich Rechtswissenschaft, Freie Universität Berlin, Van’t-Hoff-Str. 8, 14195 Berlin, Deutschland

## Abstract

Der Einsatz von Künstlicher Intelligenz (KI) im Versicherungssektor ist in jüngerer Zeit immer mehr ins Blickfeld geraten. Im medizinischen Bereich, auf dem hier der Schwerpunkt liegen soll, gilt dies nicht allein für originär medizinische Themen wie diagnostische und therapeutische Maßnahmen. Vielmehr gewinnt KI auch in den verschiedenen Stadien des Versicherungsverhältnisses – von der Risikoeinschätzung und der Tarifierung über die Vertragsanbahnung bis hin zur Regulierung von Versicherungsfällen – zunehmend an Bedeutung. Der Einsatz von KI eröffnet viele Chancen; dabei sieht sich der Anwender allerdings auch vor einige rechtliche und ethische Herausforderungen gestellt.

## Abstract (en)

The use of artificial intelligence (AI) in the insurance sector has recently received more and more attention. In the medical field, which is the focus here, this does not only apply to classical medical topics such as diagnostic and therapeutic measures. Rather, AI is also becoming increasingly important in the various stages of the insurance relationship–from risk assessment and pricing to contract initiation and the settlement of insurance claims. The use of AI opens up many opportunities; however, the user also faces some legal and ethical challenges.

## Überblick

Im Folgenden soll zunächst darauf eingegangen werden, was in der aktuellen wissenschaftlichen Diskussion unter KI zu verstehen ist. Sodann werden praktische Einsatzfelder und Anwendungsfälle aufgezeigt. Es schließt sich eine Betrachtung des Zivilrechts an, wobei es insbesondere um Zurechnungs- und Haftungsfragen geht. Sehr wichtig ist sodann das Datenschutzrecht; gerade die im Medizinsektor bedeutsamen Gesundheitsdaten unterliegen einem gesteigerten Schutz. Sodann werden einige Einzelthemen angesprochen, nämlich der Einsatz von KI zur Betrugsbekämpfung, die Anforderungen an die IT-Sicherheit sowie die Nachvollziehbarkeit von KI-gestützten Entscheidungen. Der Ausblick ist insbesondere der ethischen Dimension des Einsatzes von KI-Systemen gewidmet.

## Was ist Künstliche Intelligenz?

Unter dem Begriff Künstliche Intelligenz werden herkömmlich ganz verschiedene Technologien thematisiert. Zu nennen sind etwa sog. *Expertensysteme*, bei denen aus einer Wissensbasis regelbasiert Antworten und Handlungsempfehlungen abgeleitet werden.^1^ Hinzu kommen verschiedenste Ausprägungen von Mustererkennungssoftware, welche in großen Datenmengen Gesetzmäßigkeiten erkennt und sie für den beabsichtigten Einsatzzweck fruchtbar macht.^2^

Ein weiterer, gerade im Medizinsektor praktisch zunehmend bedeutsamer Sektor, der teils gleichfalls unter den Begriff der KI gefasst wird, ist die *Robotik*. Dabei geht es um die Ersetzung oder Ergänzung menschlicher motorischer Handlungen durch Maschinen. Ein Beispiel bietet der Fall, dass Medikamente für einen Pflegepatienten^3^ automatisiert vorsortiert werden.^4^ Das *Maschinelle Lernen* verbindet diese Teilbereiche und hebt dank enormer technologischer Fortschritte in den letzten zehn Jahren die KI-Forschung auf ein neues Level. In künstlichen neuronalen Netzen werden dabei Eingabeinformationen verarbeitet, sodass mithilfe von vielen aufeinander folgenden Trainingseinheiten und Feedbackmechanismen immer präzisere Ausgabeinformationen erzeugt werden.^5^

Dieser Output kann je nach Einsatzzweck der Technologie als Handlungsempfehlung, eigenständige Entscheidung oder reines Wissenselement gestaltet werden. Im Bereich des sog. *supervised learning* wird dabei die Erkennung von Mustern noch durch menschliches Handeln unterstützt,^6^ indem etwa Eingabedaten einem bestimmten Krankheitsbild zuordnet werden. Grundlegend anders ist das *unsupervised learning*, bei dem die KI die Daten ohne eine derartige Unterstützung eigenständig klassifiziert.^7^ Der Algorithmus ist in diesem Fall selbst in der Lage, sich zu trainieren, ohne dass er durch strukturierte Eingabeinformationen angeleitet wird.

Das Ziel des Einsatzes von KI lässt sich verallgemeinernd dahin zusammenfassen, dass es darum geht, menschliche Intelligenzleistungen entweder zu ergänzen oder sogar gänzlich zu ersetzen.^8^ Dafür braucht man viele Daten, und diese Daten müssen eine hohe Qualität aufweisen. Darauf wird noch zurückzukommen sein. Ein naheliegender Einsatzbereich von KI sind Routineentscheidungen mit vergleichbarer Entscheidungsgrundlage, also repetitive Aufgaben, die bislang von Menschen erledigt werden. Hier verspricht der Einsatz von KI nicht nur eine Kostenersparnis, sondern auch eine höhere Zuverlässigkeit und Genauigkeit.

Dies zeigt sich insbesondere bei der Tiefenanalyse von unstrukturierten Daten. Hierbei werden etwa Tonaufnahmen, Videos, Bilder oder große Textmengen in strukturierte Daten umgewandelt und auf Gesetzmäßigkeiten und Abweichungen davon untersucht. Im Vordergrund steht das Ziel, bessere Ergebnisse zu erzielen, als wenn die menschlichen Sinne allein eingesetzt würden. Eine mögliche Ausgabeinformation im medizinischen Bereich kann dann etwa in der Mitteilung bestehen, ob auf dem Bild ein Tumor oder aber gesundes Gewebe zu sehen ist.

Worin bestehen nun die mit dem Einsatz von KI verbundenen Risiken? Je komplexer die eingesetzte KI-Technologie ist, umso größer wird das Risiko von Fehlentscheidungen. Hinzu kommt die Gefahr von Sicherheitslücken, die etwa durch Cyberattacken ausgenutzt werden könnten. Dabei geht es nicht nur um mögliche Erpressungsversuche, sondern auch um Manipulationen der eingesetzten Algorithmen. Ein weiteres Thema ist der Vorwurf der Diskriminierung durch KI-gestützte Entscheidungen. Hinzu kommt die Gefahr, dass datenschutzrechtliche Vorgaben verletzt werden. Hierzu sind durch die europäische Datenschutzgrundverordnung (DSGVO) scharfe Sanktionen eingeführt worden. Nicht zuletzt ist der Einsatz von KI auch ein Thema für die Versicherungsaufsicht. Dies gilt insbesondere im Hinblick auf das Gebot der Nachvollziehbarkeit, wenn durch KI Entscheidungen getroffen werden. Führt z. B. eine KI-gestützte Risikoanalyse dazu, dass ein Versicherungsantrag abgelehnt wird oder eine Regulierungsentscheidung für den Versicherungsnehmer (oder auch – bei sachwidriger Überkompensation des Schadens – für das Kollektiv) ungünstig ausfällt, so ist dies auch ein Thema für die BaFin.

## Anwendungsfelder

Im *Medizinsektor* wird KI bereits heute in Diagnostik und Therapie auf vielen Feldern eingesetzt. Beispiele bilden das Monitoring von Körperfunktionen oder die Tumorerkennung. Die KI-gestützte Auswertung von Röntgenaufnahmen kann Fehler aufgrund menschlicher Faktoren, etwa die Fehldeutung eines Schattens oder Konzentrationslücken, vermeiden. Zudem ermöglicht der Abgleich mit einer enormen Zahl von Daten eine präzisere Diagnostik und darauf gestützte Behandlungsvorschläge. So hat eine Studie zu einem selbstlernenden KI-System zur Tumorerkennung ergeben, dass es treffsicherer war als die menschliche Diagnose durch 136 von 157 Dermatologen.^9^

Damit wird das menschliche Auge in der Diagnostik keineswegs entbehrlich, sein Einsatz kann aber für die kritischen Zweifelsfälle reserviert und insgesamt durch KI ergänzt werden. So können diagnostische Prozesse beschleunigt und ihre Qualität durch eine Kombination menschlicher und künstlicher Diagnose gesteigert werden. Im Bereich der sog. personalisierten Medizin lässt sich KI einsetzen, um anhand von Daten wie dem Körpergewicht, dem Alter oder historischer Behandlungsdaten anderer Patienten geeignete Behandlungsstrategien zu entwerfen. Ein weiteres Einsatzfeld von KI-gestützten Anwendungen bildet die Nutzung von Robotern im Pflege‑, Palliativ- und Hospizbereich, mit oder ohne Patientenkontakt.^10^

Bei *Versicherern* und damit auch im Bereich der Personenversicherung kommt KI gleichfalls mehr und mehr zum Einsatz. So nimmt sie eine wichtige Rolle bei der sog. Dunkelverarbeitung ein. Dabei geht es darum, Standardvorgänge wie die Bearbeitung unproblematischer Anträge oder Belege in der Lebens- und Krankenversicherung so weitgehend zu automatisieren, dass kein Mensch mehr mit den Vorgängen befasst wird. Die Unterlagen werden digitalisiert dem KI-Algorithmus überlassen, der eine Entscheidung trifft, die dann so umgesetzt wird. Freilich müssen die Versicherer dabei mit dem Risiko von Fehlentscheidungen rechnen, etwa wenn eine Regulierung nicht oder nur in geringerem Umfang hätte erfolgen dürfen. Die daraus erwachsenden Nachteile dürften aber zum einen durch die erhebliche automatisierungsbedingte Kostenersparnis kompensiert werden. Zum anderen ist durch die Dunkelverarbeitung auch eine höhere Kundenzufriedenheit wegen schnellerer Bearbeitungszeiten zu erwarten, was freilich ebenfalls positive ökonomische Auswirkungen mit sich bringt.

Ein weiteres Einsatzfeld ist der Vertrieb von Versicherungsprodukten. So lässt sich durch den Einsatz von KI in gewissem Umfang ermitteln, wo Versicherungsnehmer vielleicht einen Bedarf auch in anderen Bereichen haben (*cross-selling*, bei höherwertigen Versicherungsprodukten: *up-selling*) oder wo sie eine Kündigung erwägen (*churn rate*). Im Vertragsanbahnungsstadium kann die KI die von Versicherern und Vermittlern gem. §§ 6 Abs. 1, 61 Abs. 1 VVG geschuldete bedarfsgerechte Beratung unterstützen, insbesondere im Hinblick auf die Ermittlung des jeweiligen individuellen Bedarfs.

Mittlerweile ist auch der Einsatz von KI zur Erkennung von Depressionserkrankungen technisch möglich. Bedeutsam können solche Informationen insbesondere im Zusammenhang mit der Risikoprüfung vor der Antragsannahme sein, womöglich aber auch nach Anzeige eines Versicherungsfalls. Mittels KI lässt sich das Verhalten eines Versicherungsnehmers in sozialen Netzwerken wie Facebook oder Twitter analysieren; daraus schließt der Algorithmus sodann auf den Gemütszustand und auf eine mögliche Depressionserkrankung dieser Person.^11^ Dasselbe gilt für die Auswertung von Stimmen, also etwa in Telefongesprächen oder Videokonferenzen. Insoweit bestehen allerdings erhebliche Bedenken in rechtlicher und ethischer Hinsicht, auf die noch einzugehen sein wird.

## Haftungsrechtliche Fragen

### Zurechnung von KI-Verhalten

Im Zivilrecht stellen sich im Zusammenhang mit dem Einsatz von KI einige Fragen. Eine zentrale vertrags- und haftungsrechtlich bedeutsame Thematik betrifft die Zurechnung von durch KI erzeugtem Handeln. Teils wird vertreten, dass KI-Systeme als E‑Person, also als eine eigenständige rechtsfähige Einheit mit Haftungsfähigkeit anzusehen sind oder künftig durch die Rechtordnung entsprechend ausgestattet werden sollten.^12^ Diese Konstruktion begegnet aus mehreren Gründen durchgreifenden Einwänden. Zunächst fehlt es im geltenden Recht an einer Rechtsgrundlage, sodass die Rechtsfähigkeit sich allein durch den Gesetzgeber für die Zukunft einführen ließe. Eine solche Regelung würde indes das Ziel, ein Haftungssubjekt für durch KI-Systeme verursachte Schäden zu schaffen, verfehlen, solange solche Systeme nicht mit einer eigenen Haftungsmasse ausgestattet sind. Wer diese Vermögensmasse aufbringen sollte, ist indessen völlig offen.

Für die Einführung einer E‑Person besteht darüber hinaus auch gar kein Bedarf. Vielmehr gelingt es, mit dem klassischen Instrumentarium des Zivilrechts zu sachgerechten Ergebnissen zu gelangen:^13^ Wer die KI in seinem persönlichen Interesse einsetzt, um eigene Handlungen zu unterstützen oder zu ersetzen, dem sind grundsätzlich auch die Verhaltensweisen, etwa die Äußerungen der KI-Systeme zuzurechnen. Wer KI-Systeme einsetzt, hat durch die zur Verfügung gestellten Daten und das durchgeführte Training einen stets im Output erkennbaren Einfluss auf das KI-Verhalten. Bei dadurch automatisiert erzeugten Willenserklärungen ist die Zurechnung zum Verwender ohne Weiteres über den stets bestehenden Handlungswillen bei der Einschaltung vorzunehmen. Dabei dürfte in den meisten Fällen auch ein Rechtsbindungswille zu bejahen sein.^14^

In Fällen vertraglicher und deliktischer Haftung besteht indes Streit zu der Frage, ob eine Zurechnung über die §§ 278, 831 BGB analog erfolgen kann, oder ob ein eigenes Verschulden des Verwenders im Rahmen der Verletzung von Sorgfalts- und Verkehrssicherungspflichten maßgeblich ist.^15^ In diesem Bereich wird für das Jahr 2022 mit Spannung ein Legislativvorschlag der EU-Kommission zur Regelung der zivilrechtlichen Haftung erwartet, der sogar auf eine Gefährdungshaftung hinauslaufen könnte.

Die Lösung des Streits hängt letztlich davon ab, ob man KI selbst in ihrer Heterogenität als eine so gefährliche Technologie begreift, dass eine verschuldensunabhängige Haftung erforderlich ist. Denn dies wäre neben der potenziellen Einführung einer Gefährdungshaftung auch die faktische Folge einer Zurechnung des KI-Verhaltens analog § 278 BGB. Einigkeit besteht bei Vertretern beider Auffassungen jedenfalls darin, dass Haftungslücken zulasten der Betroffenen vermieden werden müssen.

### Behandlungsvertrag

Für den medizinischen Behandlungsvertrag schreibt § 630a Abs. 2 BGB vor, dass die Behandlung nach den im Behandlungszeitpunkt bestehenden allgemein anerkannten fachlichen Standards zu erfolgen hat, sofern keine abweichende Vereinbarung getroffen ist. Zu diesen Standards gehört es beim Einsatz von KI-Software, dass diese als Medizinprodukt i. S. v. Art. 2 Nr. 1 der europäischen Medizinprodukteverordnung^16^ zugelassen ist. Darüber hinaus muss künftig nach dem Entwurf der EU-Kommission zu einer KI-Verordnung^17^ ein umfangreicher Pflichtenkatalog auch und gerade beim Einsatz von medizinischer KI-Software eingehalten werden.

Nach § 630c Abs. 2 BGB sind dem Patienten zu Beginn der Behandlung sämtliche für die Behandlung wesentlichen Umstände zu erläutern. Zu diesen Umständen zählt auch der Einsatz von KI-Systemen. Dasselbe gilt hinsichtlich der im Vorfeld der Einwilligung in den ärztlichen Heileingriff erforderlichen Aufklärung, die sich gem. § 630e Abs. 1 BGB auf sämtliche für die Einwilligung des Patienten wesentlichen Umstände zu erstrecken hat.

### Verhältnis von Arzthaftung und Herstellerhaftung

Eine interessante Frage stellt sich im Zusammenhang mit der Haftung für KI-Systeme im Hinblick auf das Verhältnis von Arzthaftung und Herstellerhaftung. Es geht darum, ob der Einsatz von KI zu einer Verlagerung von der Arzthaftung hin zur Haftung der Hersteller von KI-Systemen führt. Für die Bereiche des hochautomatisierten und autonomen Fahrens wird teils Entsprechendes, nämlich eine Verschiebung von der Kfz-Halterhaftung hin zur Herstellerhaftung, angenommen.^18^

Gegen eine derartige Verlagerung spricht im hier interessierenden Kontext, dass es auch beim Einsatz von KI-Systemen weiterhin der Arzt ist, der die Entscheidung für eine bestimmte Behandlung und deren Durchführung trifft. Die KI verdrängt ihn nicht aus dieser Stellung und der damit verbundenen, soeben geschilderten haftungsrechtlichen Verantwortung. Eine Verlagerung der Haftung findet dadurch zwar nicht statt. Allerdings wird auch der Hersteller bei Fehlern der KI nicht gänzlich aus seiner Verantwortung entlassen. In Betracht kommen insoweit die deliktsrechtliche Produzentenhaftung, die Produkthaftung nach dem ProdHaftG und ggf. Gewährleistungsansprüche des Verwenders.

### Haftpflichtversicherungsschutz nach den AVB Arzt

Inwieweit besteht für die Haftung für Schäden, die durch fehlerhafte KI-Systeme verursacht worden sind, Versicherungsschutz? In diesem Kontext stellt sich unter dem Schlagwort „Silent AI“ die Frage, ob derartige Haftungsrisiken aus dem Einsatz von KI vom Deckungsumfang einer Haftpflichtversicherung umfasst sind. Nach Nr. A1‑1.2. AVB Arzt^19^ besteht Versicherungsschutz für die gesetzliche Haftpflicht des Arztes „aus Behandlungen und Besitz und Verwendung von Apparaten ausschließlich, soweit die Behandlungen und Apparate in der Heilkunde anerkannt sind“. Diese sog. Apparateklausel erfasst beispielsweise Roboter, etwa Pflegeroboter.

Ungeklärt ist allerdings die Frage, ob zu den Apparaten auch eine Software zählt, die nicht in die für die Behandlung bedeutsamen physischen Elemente implementiert ist. Dagegen spricht, dass es insoweit um reine Softwareanwendungen in Computern geht (z. B. Diagnosesoftware) und nicht um die Anerkennung eines Computers als „Apparat“ in der Heilkunde. Hinzu kommt, dass im Bereich von Software auch die Zulassung als Medizinprodukt erforderlich ist (s. oben sub 4.2).

### Haftung des Versicherers bei eigenem KI-Einsatz

Der Versicherer haftet grundsätzlich nach den allgemeinen Regeln für Handlungen der durch ihn eingesetzten KI. Dies gilt etwa bei Diskriminierungen in der Preisfindung oder bei Datenschutzverstößen. Besonderheiten ergeben sich bei der Ersetzung menschlicher Beratungsleistungen. Dies ist insbesondere dann der Fall, wenn der Versicherer KI im Rahmen der vorvertraglichen Beratung z. B. in Gestalt von Chat- oder gar Voicebots einsetzt. Mittlerweile ist diese Technik der Simulation eines menschlichen Gesprächs so weit fortgeschritten, dass es selbst Fachleuten bisweilen schwer fällt zu unterscheiden, ob ein Mensch spricht bzw. schreibt oder ein sehr gut trainiertes KI-System. Ist an einer Kommunikation zwischen Versicherungsnehmer und Versicherer oder Vermittler kein Mensch mehr beteiligt ist, dann muss (neben den bedarfsbezogenen Beratungspflichten gem. §§ 6 Abs. 1, 61 Abs. 1 VVG) darauf vorab hingewiesen werden, dass er es ausschließlich mit einem KI-gesteuerten Gegenüber zu tun hat. Diese Schutzpflicht ergibt sich schon aus § 241 Abs. 2 BGB. Sie findet sich aber auch als Informationspflicht in Art. 52 des Verordnungsentwurfs der EU-Kommission^20^ zur Regulierung von KI wieder.

Diese Regelung ist im Übrigen die einzige Norm des Kommissionsvorschlags, welche Versicherer als Verwender von nicht verkörperter KI-Software betrifft. Das vornehmlich produktebezogene Regelwerk hat daher kaum Auswirkungen auf den Einsatz von KI im Versicherungswesen, umso mehr jedoch für den Medizinsektor, in dem Produkte eine bedeutende Rolle spielen. Der Kommissionsvorschlag bleibt damit zurecht hinter der vorangegangenen Entschließung des EU-Parlaments zurück, derzufolge pauschal alle durch Versicherer angewendeten KI-Systeme als hohes Risiko eingestuft werden. Indes hat nunmehr der Rat der EU einen modifizierten Vorschlag vorgelegt, nach welchem jedenfalls die wesentlichen Bestandteile des Versicherungsgeschäfts – Prämienkalkulation, Vertragsabschluss und Schadensmanagement – von der Hochrisiko-Definition erfasst sein sollen.^21^

## Datenschutzrecht

### Grundregeln

Die unionsweit unmittelbar anwendbare DSGVO legt ein sehr weites Verständnis sowohl von personenbezogenen Daten als auch von deren Verarbeitung zugrunde.^22^ Entscheidend dafür, ob es sich um personenbezogene Daten handelt, ist letztlich stets, ob eine natürliche Person identifizierbar ist. Dies kann anhand des Namens oder einer Kennnummer der Fall sein, aber auch aufgrund von Standortdaten oder biometrischen Daten wie Gesichtserkennung oder Fingerabdrücken. Auch die menschliche Stimme genügt als Identitätsmerkmal.

Dass auch der Begriff der Verarbeitung weit verstanden wird, hat unmittelbare Auswirkungen auf den Einsatz von KI-Systemen. Grundsätzlich erfordert jede Verarbeitung personenbezogener Daten eine Einwilligung des Betroffenen. Allerdings ist sie in bestimmten Fällen nicht notwendig, etwa wenn die Datenverarbeitung zur Vertragsdurchführung erforderlich ist. Die besonderen Zulässigkeitsgründe sind in den Art. 6, 9 und – diese ergänzend – 22 DSGVO geregelt. Wenn für KI-Anwendungen auf bereits für andere Zwecke erhobene Daten zurückgegriffen wird, sind insbesondere die Regeln des Art. 6 Abs. 4 DSGVO zu beachten. In diesem Kontext ist angesichts des risikobasierten Ansatzes der Verordnung nach Art. 6 Abs. 4 lit. c DSGVO insbesondere die Art der personenbezogenen Daten zu beachten. Dabei geht es vor allem darum, ob die Verarbeitung von gesundheitsbezogenen Daten gem. Art. 9 DSGVO erfolgt (s. dazu noch sogleich sub 5.2). Je größer das mit der Datenverarbeitung einhergehende Risiko von Verstößen ist, umso strenger sind die Anforderungen an die Vereinbarkeit der neuen Verarbeitungszwecke mit den ursprünglich verfolgten Zwecken.^23^

### Insbesondere gesundheitsbezogene Daten

Ein besonders hohes Schutzniveau ist für *gesundheitsbezogene* und damit besonders sensible Daten vorgesehen. Ihnen widmet nicht allein das VVG in seinem § 213 eine eigene Regelung, sondern auch die DSGVO in Art. 4 Nr. 15 (Definition) und Art. 9 Abs. 2 (Entbehrlichkeit der Einwilligung). Für die Verarbeitung gesundheitsbezogener Daten bedarf es grundsätzlich einer ausdrücklichen Einwilligung und es gibt im Vergleich zu Art. 6 DSGVO weniger Ausnahmefälle, in denen das nicht der Fall ist. Dabei geht es insbesondere um die Situation, dass die Informationen nach Anzeige eines Versicherungsfalls zur Schadensbearbeitung erforderlich sind. Die Datenverarbeitung ist dann gem. Art. 9 Abs. 2 lit. f DSGVO zulässig. Der Versicherungsnehmer kann die Verarbeitung der für die Schadensregulierung relevanten Daten nicht verhindern, indem er seine Einwilligung verweigert.

Unter besonderem Schutz steht die Selbstbestimmung des Betroffenen über seine genetischen Daten. Das Gendiagnostikgesetz untersagt in seinem § 18 Abs. 1 S. 1 Nr. 2 grundsätzlich bereits die Entgegennahme von Ergebnissen oder Daten aus bereits vorgenommenen genetischen Untersuchungen oder Analysen, selbst wenn ein Versicherungsnehmer sie dem Versicherer geradezu aufdrängen möchte, um zu belegen, dass er eine günstige Risikoprognose aufweist.^24^ Lediglich für bestimmte Personenversicherungen mit höheren Summen gilt hiervon eine Ausnahme (§ 18 Abs. 1 S. 2 GenDG); zudem wird das Verbot dadurch relativiert, dass die Gesundheitsfragen im Rahmen der vorvertraglichen Anzeigepflicht nach § 19 VVG sich auch auf solche Vorerkrankungen und Erkrankungen erstrecken dürfen, die mittels eines genetischen Tests diagnostiziert worden sind (§ 18 Abs. 2 GenDG). § 18 Abs. 1 GenDG soll nämlich nur das Recht auf Nichtwissen schützen; bereits bekannte Erkrankungen sind anzuzeigen.^25^

Bisweilen stellt sich die Frage, ob es sich bei bestimmten Daten überhaupt um gesundheitsbezogene Daten handelt, also um solche Daten, die sich auf die Gesundheit beziehen und aus denen Informationen über den Gesundheitszustand hervorgehen. Dies gilt etwa für den bereits in anderem Zusammenhang erwähnten Fall, dass ein KI-System das Verhalten des Versicherungsnehmers in sozialen Netzwerken analysiert, um daraus Folgerungen für das Risiko von Depressionen zu ziehen.

Gegen die Einordnung als Gesundheitsdaten ließe sich hier anführen, dass es sich bei den verarbeiteten Daten nicht um solche handelt, aus denen unmittelbar Informationen über den Gesundheitszustand hervorgehen; solche Informationen werden vielmehr erst durch die KI generiert. Allerdings spricht der Zweck der Verarbeitung, Depressionserkrankungen zu erkennen, dafür, auch insoweit von Gesundheitsdaten auszugehen. Dementsprechend wird auch im Schrifttum vertreten, dass insoweit, als aus anderen Daten mittelbar Rückschlüsse auf den Gesundheitszustand eines Betroffenen gezogen werden können (oder dies vermutet wird), bereits diese Daten vom Begriff der Gesundheitsdaten umfasst sind.^26^ Generell dürfte davon auszugehen sein, dass der Europäische Gerichtshof die Vorgaben der DSGVO im Sinne des vom europäischen Verordnungsgeber angestrebten starken Datenschutzniveaus weit auslegen wird.

## Betrugsbekämpfung

Ein wichtiges Anwendungsfeld von KI-Systemen ist die Betrugsbekämpfung. Dabei ist die Betrugsaufdeckung dadurch, dass die Stimme dessen, der einen Schaden meldet, nach Art eines modernen Lügendetektors analysiert wird, datenschutzrechtlich problematisch. Die Datenverarbeitung ist nämlich regelmäßig für die in Art. 6 Abs. 1 lit b–e DSGVO genannten Zwecke nicht erforderlich. Wird die Verarbeitung auf Art. 6 Abs. 1 lit. f DSGVO gestützt, so muss eine Interessenabwägung stattfinden. Diese wird regelmäßig nicht zur Zulässigkeit führen. Nach Erwägungsgrund 47 der DSGVO stellt die Verarbeitung personenbezogener Daten im für die Verhinderung von Betrug unbedingt erforderlichen Umfang ein berechtigtes Interesse des jeweiligen Verantwortlichen dar. Allerdings ist die Verarbeitung auch dann, wenn die Voraussetzung der Erforderlichkeit erfüllt ist, ausgeschlossen, wenn dem überwiegende Interessen oder Grundrechte und Grundfreiheiten des Betroffenen entgegenstehen.^27^

Ein unbedenkliches Einsatzfeld von KI ist hingegen die Plausibilitätsprüfung von eingereichten Belegen. Hierzu gibt es in der Praxis bereits zahlreiche KI-gestützte Verfahren, die dem Versicherer eine effektivere Dunkelverarbeitung bei gleichzeitiger Aussteuerung auffälliger Belege ermöglichen.

## Aufbau größerer Datenbanken

KI-Systeme erfüllen die ihnen zugedachten Aufgaben umso zuverlässiger, je mehr und je hochwertigere Daten sie verarbeiten können. Wenn jeder Versicherer die Daten nur für sich sammelt und auswertet, wird der Datenbestand oft unzureichend sein; dies gilt umso mehr für neu auf einen Markt tretende Anbieter. Es erscheint daher erwägenswert, auf deutscher oder gar auf europäischer Ebene eine gemeinsame Datenbank aufzubauen. Sofern die Daten anonymisiert sind, droht kein Konflikt mit den Vorgaben der DSGVO. Was etwaige kartellrechtliche Bedenken angeht, so gibt es zwar keine Gruppenfreistellungsverordnung für den Versicherungssektor mehr, wohl aber den Freistellungstatbestand des § 2 GWB. Er betrifft den Fall, dass es um die Verbesserung oder Förderung des technischen Fortschritts geht. Zudem geht es bei solchen Kooperationen nicht darum, den Wettbewerb zu beschränken, sondern im Gegenteil ihn überhaupt erst zu ermöglichen.

Alternativ käme auch der Aufbau einer Datenbank durch einen öffentlichen Träger oder einen treuhänderisch tätigen privaten Datenvermittler in Betracht. Einen zusätzlichen Weg bieten sog. digitale Ökosysteme. Gelingt es dem Versicherer, Kunden in sämtlichen Lebensbereichen datenbasiert in das eigene Geschäftsmodell einzubinden, so eröffnen sich für KI-Anwendungen deutlich weiterreichende Möglichkeiten.

## IT-Sicherheit

Geht es um IT-Sicherheit beim Versicherer, ist das Vertrauen der Versicherungsnehmer ein zentrales Thema. Insoweit steht eine neue Verordnung zur Betriebsstabilität digitaler Systeme speziell im Finanzsektor bevor.^28^ Hierdurch sollen den Finanzunternehmen umfangreiche Governance-Maßnahmen auferlegt werden, um sämtliche geschäftsrelevanten IT-Infrastrukturen hinreichend gegen unbefugtes Eindringen sowie Schädigungen von Innen zu sichern.

Darüber hinaus schreibt der Kommissionsentwurf einer KI-Verordnung^29^ ein hohes Maß an „Robustheit“ und Cybersicherheit von KI-Systemen vor. Dabei wird der Begriff der „Robustheit“ in Erwägungsgrund 50 des Kommissionsvorschlags definiert als Widerstandsfähigkeit gegen Fehler, Störungen, Manipulationen und unerwartete Situationen. Schon jetzt unterliegen Unternehmen, die KI-Systeme einsetzen, gewissen Verkehrssicherungspflichten hinsichtlich der IT-Sicherheit. Diese können sich etwa aus dem BSIG ergeben.^30^ Umgekehrt kann auch der Versicherer im Schadensfall prüfen, ob der Versicherungsnehmer zumindest grundlegende Sicherheitsanforderungen an schadensträchtige und versicherte IT-Produkte erfüllt hat.

In der Praxis werden entsprechende vertragliche Standards insbesondere durch die Obliegenheiten des Versicherungsnehmers in der unternehmensbezogenen Cyberversicherung gesetzt. Dabei wird hinsichtlich der Anforderungen regelmäßig zwischen Industrieunternehmen einerseits und KMU andererseits differenziert. Zugleich richtet sich der Blick angesichts des Trends hin zu smarten Endgeräten im Haushalt (smart home) und der durch die Covid-19-Pandemie intensivierten Arbeit im Homeoffice mittlerweile auch immer mehr auf Verbraucher.

## Nachvollziehbarkeit KI-generierter Entscheidungen

Ein nicht allein für die Vertrauensbildung, sondern auch für die Versicherungsaufsicht bedeutsames Thema ist die Nachvollziehbarkeit von Entscheidungen, die durch KI erzeugt werden. Je technisch ausgefeilter die KI-Systeme werden, umso schwieriger wird die Nachvollziehbarkeit der durch sie generierten Entscheidungen. Insbesondere bei selbstlernenden Systemen ist es bisher kaum im Detail nachvollziehbar, wie sie letztlich zu ihren Entscheidungen gelangt sind.

Damit die BaFin ihren Aufsichtsauftrag ordnungsgemäß erfüllen kann, muss ihr in einer objektiv nachvollziehbaren Weise erläutert werden, wieso bestimmte Antragsablehnungs- oder Regulierungsentscheidungen erfolgt sind. Die Behörde hat bereits verlautbart, dass sie solche Algorithmen nicht akzeptieren wird, bei denen die Nachvollziehbarkeit unzureichend erscheint. Schon im Jahr 2018 hat sich die BaFin zu der Thematik positioniert.^31^

Ein anderer Aspekt der Nachvollziehbarkeit ist, wie erwähnt, das Vertrauen der Betroffenen. Von den Befragten einer in Deutschland, den USA, Kanada, Großbritannien und Australien durchgeführten Studie vertrauen demnach nur 28 % KI im Allgemeinen.^32^ Dem halten Informatiker, die KI-Systeme entwickeln und betreuen, entgegen, dass die Fehlerquote in der Praxis sehr gering ist. Mithin entspringen die Vorbehalte gegen den Einsatz von KI-Systemen offenbar eher einem diffusen, nicht rational erklärbaren Misstrauen. Dem wird sich in gewissem Maße entgegenwirken lassen, indem die Nachvollziehbarkeit verbessert wird. Dies gelingt vor allem durch ein hohes Maß an Datenqualität und Trainingsaufwand, um Fehler zu minimieren und ein besseres Verständnis der Technologie zu erlangen. Zudem ist es unabdingbar, Transparenz gegenüber den durch den Einsatz von KI-Systemen Betroffenen zu schaffen und zu erklären, wie die KI-Systeme aufgebaut sind und wie sie funktionieren.

## Ausblick, insbesondere zur ethischen Dimension

Unter dem Schlagwort „*Entmenschlichung*“ wird der Einsatz von Robotern etwa im Bereich der Pflege in der Öffentlichkeit häufig kritisiert. Der Tenor dieser Kritik lautet, dass anstelle der persönlichen Ansprache durch einen Menschen die Maschine tritt, die emotionslos und distanziert technische Verrichtungen ausführt. Dabei wird häufig übergangen, dass dann, wenn Routineaufgaben durch KI-Einsatz erledigt werden, die Pflegekräfte mehr Zeit für die menschliche Zuwendung haben, die derzeit vielfach zu kurz kommt.

Der Einsatz von KI-Systemen birgt auch die Gefahr, dass es zu nach dem AGG unzulässigen *Diskriminierungen* kommt. Dazu kann es dann kommen, wenn das System fehlerhaft programmiert ist oder falsch angelernt wird. Dabei ist es wichtig die mitunter strengen Anforderungen des europäischen Gleichbehandlungsrechts umzusetzen. Ein Beispiel bietet das Verbot geschlechtsbezogener Differenzierungen bei Prämien und Leistungen (§ 19 Abs. 1 Nr. 2, 20 Abs. 2 AGG). Wird die KI so trainiert, dass sie Daten auch anhand des Geschlechts der betreffenden Personen auswertet, gelangt sie alsbald zu der Erkenntnis, dass die Lebenserwartung von Frauen signifikant höher liegt als diejenige von Männern, und dass jeweils teils geschlechtsbezogen unterschiedliche Krankheitsbilder zu verzeichnen sind. Auch wenn diese Unterschiede bei einer rein aktuariellen Betrachtung zu entsprechend verschiedenen Risikobewertungen und infolgedessen Vertragskonditionen führen müssten, hat der EuGH entsprechende Differenzierungen rundheraus untersagt.^33^ Derartige für die Praxis verbindliche rechtliche Schranken müssen bei der Programmierung der KI-Algorithmen beachtet werden.

Als ein allgemeines, anwendungsübergreifendes ethisches Gebot lässt sich die Forderung aufstellen, dass der Einsatz von KI-Systemen stets einen *Mehrwert für die betroffenen Patienten und Versicherungsnehmer* bieten sollte. Ein solcher Mehrwert mag auch in der Einsparung von Kosten liegen, da dies letztlich auch dem Einzelnen zugutekommt, etwa über niedrigere Versicherungsprämien und ggf. auch Behandlungskosten. Gerade in der Medizin sollte der KI-Einsatz jedoch möglichst einen Mehrwert haben, der über eine risikoadäquatere Prämiengestaltung hinausgeht, indem er gesundheitsbezogen ist, sei es in Gestalt einer zuverlässigeren Diagnostik oder verbesserter Therapiemöglichkeiten. Die Entscheidung, KI nicht lediglich zur Kostensenkung, sondern vor allem zur Qualitätssteigerung einzusetzen, dürfte sich regelmäßig als nachhaltiger erweisen.

Ein weiteres ethisches Postulat lautet, dass der menschliche Faktor gerade im Medizinsektor durch den Einsatz von KI-Systemen nicht komplett ersetzt, sondern lediglich ergänzt werden sollte. Teils ist dies ohnehin jedenfalls derzeit noch aus technischen Gründen geboten. So gibt es in der Radiologie immer wieder Fälle, bei denen der Befund so ungewiss ist, dass die KI ein Signal gibt, damit ein Radiologe sich die Aufnahme ansieht. Ähnliches gilt beim Einsatz von KI zur Betrugserkennung. Auch Kulanzentscheidungen des Versicherers, welche ein menschliches Element in die Beziehung zum Versicherungsnehmer einbringen, sollten künftig nicht in vollem Umfang der KI überlassen werden, auch wenn sie selbst dieses von Ermessensspielräumen geprägte Feld mittlerweile in weitem Umfang nachbilden kann.

## Footnotes

1 *Kaplan*, Künstliche Intelligenz – Eine Einführung, Übersetzung v. Guido Lenz, 2017, S. 38 f.; *Stiemerling*, in: Kaulartz/Braegelmann, Rechtshandbuch Artificial Intelligence und Machine Learning, 2020, Kapitel 2.1 Rn. 35. *Stiemerling* (Fn. 2), Kapitel 2.1 Rn. 7 ff.

2 *Stiemerling* (Fn. 2), Kapitel 2.1 Rn. 7 ff.

3 Aus sprachlichen Gründen wird in diesem Text das generische Maskulinum verwendet; die Ausführungen gelten freilich gleichermaßen für alle Geschlechter.

4 *Brand*, MedR 2019, 943.

5 *Niederée/Nejdl*, in: Ebers/Heinze/Krügel/Steinrötter, Rechtshandbuch Künstliche Intelligenz und Robotik, 2020, § 2 Rn. 24 ff., 56 ff.

6 *Niederée/Nejdl* (Fn. 5), § 2 Rn. 40.

7 *Niederée/Nejdl* (Fn. 5), § 2 Rn. 41.

8 Siehe die Beispiele in *Günther/Böglmüller*, BB 2017, 53, 56 f.

9 *Brinker/Hekler et al.*, European journal of cancer 2019, Vol. 113, 47 ff.

10 Dazu näher *Beck*, MedR 2018, 772 ff.

11 *Frost*, MPR 2019, 117, 118.

12 S. nur *Schirmer*, JZ 2016, 660 ff.; *Teubner*, AcP 2018, 155, 182.

13 So auch u. a. *Foerster*, ZfPW 2019, 418, 426 ff., 430 ff.; *Gitter*, Softwareagenten, S. 181 f.; *Riehm*, in: Kaulartz/Braegelmann (Fn. 2), Kapitel 6.1 Rn. 44 ff.

14 Vgl. *Nitschke*, Verträge unter Beteiligung von Softwareagenten – ein rechtlicher Rahmen, 2010, S. 81.

15 Für die Analogien etwa *Hacker*, RW 2018, 243, 249 ff., 265 ff.; *Keßler*, MMR 2017, 589, 592 f.; dagegen z. B. *Foerster*, ZfPW 2019, 418, 431; *Freyler*, NZA 2020, 284, 289; *Horner/Kaulartz*, CR 2016, 7; *Klingbeil*, JZ 2019, 718, 720.

16 VO (EU) 2017/745.

17 COM (2021) 206 final.

18 S. etwa Balke, SVR 2018, 5, 7; zur Möglichkeit einer faktischen Verlagerung *Armbrüster*, in: Gless/Seelmann, Intelligente Agenten und das Recht, 2016, S. 205, 218.

19 Musterbedingungen des GDV, Stand: Mai 2020; abrufbar unter: www.gdv.de.

20 COM (2021) 206 final.

21 2021/0106(COD), S. 98. Vorausgehend: Entschließung des EU-Parlaments v. 20.10.2020, P9_TA (2020) 0276; https://versicherungswirtschaft-heute.de/maerkte-und-vertrieb/2020-11-23/eu-parlament-stuft-einsatz-von-ki-bei-versicherern-als-hochriskant-sein/, zuletzt aufgerufen am 25.07.2022, 17.22 Uhr.

22 *Ernst*, in: Paal/Pauly DSGVO BDSG, 3. Aufl. 2021, Art. 4 DSGVO Rn. 3, 20.

23 *Spindler/Dalby*, in: Spindler/Schuster, Recht der elektronischen Medien, 4. Aufl. 2019, Art. 6 DSGVO Rn. 28.

24 *Fenger*, in: Spickhoff, Medizinrecht, 3. Aufl. 2018, § 18 GenDG Rn. 4.

25 BT-Drucks. 16/10532, S. 36.

26 *Ernst*, in: Paal/Pauly, DSGVO BDSG, 3. Aufl. 2021, Art. 4 DSGVO Rn. 108.

27 *Frenzel*, in: Paal/Pauly, DSGVO BDSG, 3. Aufl. 2021, Art. 6 DSGVO Rn. 30.

28 COM (2020) 595 final.

29 COM (2021) 206 final.

30 *Riehm/Meier*, MMR 2020, 571.

31 *BaFin*, Big Data trifft auf künstliche Intelligenz, 2018, S. 13.

32 https://home.kpmg/de/de/home/themen/2021/05/studie-buergerinnen-und-buerger-haben-wenig-vertrauen-in-ki.html, zuletzt aufgerufen am 25.07.2022, 17.23 Uhr.

33 EuGH, Urt. v. 01.03.2011 – C‑236/09, NJW 2011, 907.

